# It is not worth postponing frozen embryo transfers after oocyte pickup: A retrospective cohort study based on propensity score matching

**DOI:** 10.3389/fendo.2022.971616

**Published:** 2022-09-05

**Authors:** Mengxia Ji, Bihui Jin, Xiaoyan Guo, Ruifang Wu, Yunqing Jiang, Ling Zhang, Jing Shu

**Affiliations:** Department of Reproductive Endocrinology, Center for Reproductive Medicine, Zhejiang Provincial People’s Hospital, Hangzhou Medical College, Hangzhou, China

**Keywords:** Frozen embryo transfer, freeze-all, *in vitro* fertilization, live birth, propensity score

## Abstract

This study was to explore whether postponing frozen embryo transfers (FET) after oocyte pickup (OPU) improves clinical and neonatal outcomes. From May 2018 to Dec 2020, a total of 1109 patients underwent their first OPU cycles adopting a non-selective freeze-all policy were included in this retrospective cohort study. In the immediate group (n=219), patients underwent FET in the first menstrual cycle after OPU, and patients in the postponed group (n=890) waited for more than 1 menstrual cycle after OPU to perform FET. A propensity score matching (PSM) model was used to evaluate the clinical outcomes and neonatal outcomes between the two groups. There were 209 patients in the immediate group and 499 patients in the postponed one after PSM. Patients waited for a significantly shorter period for FET in the immediate group (30.74 ± 3.85 days) compared with the postponed group (80.39 ± 26.25 days, P<0.01). The clinical pregnancy rate (CPR) and live birth rate (LBR) in the immediate group were 58.4% and 48.3%, respectively, which were comparable to those of the postponed one (58.1%, 49.7%, P > 0.05). No statistical significance was found in the average birth weight (3088.82 ± 565.35 g vs 3038.64 ± 625.78 g, P > 0.05) and height (49.08 ± 1.87 cm vs 49.30 ± 2.52 cm) of neonates between the two groups. The gender ratio, the incidence of macrosomia and low birth weight did not differ significantly between the two groups. In conclusion, postponing FET does not improve clinical and neonatal outcomes. If patients have no contraindications, FETs should be carried out immediately after OPU.

## Introduction

In China, nearly 25% of couples in the reproductive age suffer from infertility (1), and a growing number of people are turning to reproductive centers for medical help. Consequently, the number of *in vitro* fertilization (IVF) cycles has experienced a rapid growth, accounting for 1 211 303 cycles from 2013 to 2016 (2). Meanwhile, the number of frozen embryo transfer (FET) cycles has also increased at a rate of 20% per year. By 2016, the number of babies born from frozen embryos had gradually exceeded that of babies born from fresh embryos (2). The recent dramatic rise of FET cycles in assisted reproductive technology may be partly attributed to the progress of vitrification (3). The window of implantation for fresh cycles is narrowed due to premature progesterone and supraphysiologic levels of estrogen (4). FET cycles circumvent this shortcoming, thus it is believed to get better endometrial receptivity (4). In randomized controlled trials comparing frozen with fresh embryo transfers, the live birth rate (LBR) was higher in FET cycles of patients with polycystic ovary syndrome (PCOS), while similar in ovulatory infertility (5). Another advantage of FET is that it reduces the incidence of ovarian hyperstimulation syndrome. These benefits make a freeze-all policy common in many infertility clinics. According to one large retrospective study, the LBR was as high as 50.74% in the first freeze-all FET cycle (6). A new debate has emerged in clinical practice about how long practitioners should wait before transferring frozen embryos after oocyte retrieval. Some studies have found that performing FETs immediately yields higher LBRs than delaying them (7–9). However, another study favored delaying FET (10). Recently, several investigations found that the interval between oocyte pickup (OPU) and FET did not affect LBR (11–13). These conflicting results may be attributed to different study designs, complex confounding factors such as controlled ovarian hyperstimulation (COH) protocols, endometrial preparation regimes, ovarian response, etc. To avoid the above bias, we adopted a propensity score matching (PSM) model to explore whether delaying FET after OPU affects clinical and neonatal outcomes.

## Methods

### Patients

From May 2018 to December 2020, 1 109 patients who started their first IVF cycles at the reproductive center of Zhejiang Provincial People’s Hospital were enrolled in our study. They were all younger than 40 years old and scheduled to undergo their first FET cycles within 6 months after OPU. Patients with abnormal karyotypes, a history of recurrent spontaneous abortions and using the GnRH-a downregulation protocols were excluded. Before starting IVF, all patients were routinely screened by transvaginal three-dimensional ultrasound, and if there was any abnormal, such as intrauterine adhesions, polyps and uterine septum, hysteroscopy was carried out to address these conditions. COH cycles would started until the uterine cavity returned to normal. Patients who underwent FET within the first menstrual cycle after OPU were defined as the immediate group, whereas those who underwent FET more than 1 menstrual cycle after OPU were defined as the postponed one.

All patients signed informed consent forms stating that the data collected during treatment could be used in research anonymously. This retrospective study was approved by the ethics committees of Zhejiang Provincial People’s Hospital (grant number:2021QT439).

### Controlled ovarian hyperstimulation protocols

The gonadotropin-releasing hormone (GnRH) antagonist, mild stimulation, and progesterone-priming ovarian stimulation (PPOS) protocols were adopted in this study. For the GnRH antagonist protocol, gonadotropin, including recombinant follicle-stimulating hormone (FSH) (GONAL-*f*; Merck Serono, Germany) or human menopausal gonadotropin (hMG) (Lizhu Pharmaceutical Factory, China) was started from day 2 of the menstrual cycle at a dose of 100 to 300 IU per day. It was adjusted according to the ovarian response and serum hormone levels. When the diameter of the leading follicles reached 14 mm, or the serum estradiol (E_2_) level was greater than 500 pg/mL, a GnRH-antagonist (cetrorelix, Merck Serono; or ganirelix, MSD) was injected subcutaneously at a daily dose of 0.25 mg until the trigger day.

For the mild stimulation protocol, clomiphene citrate (MEDOCHEMIE LTD, Cyprus) was started on day 2 at a dose of 50 to 100 mg with hMG (Lizhu Pharmaceutical Factory, China) at a dose of 75 to 150 IU per day. A GnRH-antagonist was added when serum LH was higher than 10 U/mL.

For the PPOS protocol, medroxyprogesterone acetate (MPA) (Zhejiang Xianju Pharmaceutical Co., China) was given at a daily dose of 6 to 8 mg from day 2 of the menstrual cycle. Simultaneously, hMG (Lizhu Pharmaceutical Factory, China) was given at a dose of 75 to 225 IU per day.

When the diameters of at least 2 leading follicles reached 18 mm, either recombinant human choriogonadotropin alfa (r-hCG) (Merck Serono, Germany) 250 μg or triptorelin (Ferring, Germany) 0.2 mg or a dual trigger with both r-hCG 250 μg and triptorelin 0.1 mg were applied to trigger the final oocyte maturation.

### FET cycles

Before starting the FET cycle, an ultrasound was routinely done on the 3rd day of the menstruation. In the first menstruation, the ovaries of most patients were enlarged after COH. HRT protocols were applied in these patients. Natural cycles were recommended for those whose ovaries returned to normal. When patients waited for one or more periods to start FET, the endometrium preparation protocols were determined according to the menstruation regularity and patients’ personal intentions. Thus in this study, we included hormone replacement therapy (HRT), natural cycle (NC) and ovarian stimulation (OS) protocols.

For HRT, estradiol valerate (Delpharm Lille S.A.S) was administered orally at a dose of 6 mg daily starting from day 3 of the menstrual cycle. After 7 days, patients whose endometrium was thinner than 7 mm were given transdermal estradiol gels (Besins Manufacturing) at a dose of 5 g daily. After at least 12 days’ medication, when the endometrial thickness was no longer improved, progesterone (Zhejiang Xianju Pharmaceutical Co. Ltd) was injected at a dose of 40 mg. For NC, follicular monitoring was started on days 10 to 12 of menstruation. When the diameter of the dominant follicle exceeded 14 mm, the serum hormone levels were examined every day to define the LH surge day and stopped when ovulation took place. The progesterone add-up started from the ovulation day or 2 days after the LH surge. The OS protocol was a remedy for NC if the dominant follicle was not detected by day 20 of menstruation. The hMG was administrated at a dose of 75 IU per day and gradually increased until a dominant follicle appeared. The rest of the procedure was as per the NC protocol.

No more than 2 embryos were transferred in the study groups. The progesterone priming day was defined as day 0. Blastocysts were transferred on day 5 and cleavage embryos on day 3. The cleavage embryos were morphologically assessed according to the number, size, and distribution of blastomeres, and the cytoplasmic fragmentation percentage (14). Embryos graded as 1 and 2 with 7-10 cells on day 3 were defined as top-quality embryos. The Gardner grading system was used for blastocysts (15). Those graded higher than 3BB on day 5 or 4BB on day 6 were deemed top-quality.

### Pregnancy outcome assessment

The blood hCG levels were tested 12 days after FET, and transvaginal ultrasound scans were performed 35 days after FET. A clinical pregnancy was confirmed if a gestational sac was detected. Spontaneous miscarriages in the first trimester were deemed as early miscarriages, and viable neonates delivered after 28 weeks of gestation were defined as live births. The LBR was the primary end point in our study. We followed up on the gestational age, the neonates’ gender, birth weight, height and pregnancy related complications, including gestational diabetes, pregnancy-induced hypertension, placenta previa and placental abruption.

### Statistical analysis

Because this was a retrospective study, to control for confounders, a PSM model was adopted using the nearest-neighbor matching method. Variables included in the PSM model were female and male age, body mass index (BMI), causes of infertility, AMH value, COH protocols, trigger protocols, the amount of Gn used, E_2_ levels on the trigger day, endometrial preparation regimens, as well as number, quality and stage of embryos transferred. We used PSM at a ratio of 1:4. After matching, the standardized mean difference was less than 0.1, indicating that variables between both groups be balanced. R software version 2.10.0 (R Core Team, October 2009) was used.

The statistical analysis was performed by IBM SPSS Statistics for Windows, Version 21.0. (IBM Corp., 2012). All measurement data were expressed as mean ± standard deviation. For continuous variables with normal distribution, the Student’s *t-*test was adopted. Pearson’s chi-squared test was applied for proportion comparisons between groups. A *P-*value less than 0.05 was defined as statistically significant.

## Results

The clinical outcomes of the 1109 patients who underwent their first FET cycles were analyzed. There were 219 patients in the immediate group and 890 in the postponed (see **Table 1**). The average waiting time for FET was significantly shorter in the immediate group than in the postponed one (30.77 ± 3.81 days vs. 80.72 ± 26.49 days; *P* < 0.05).

**Table 1:** Baseline characters and treatment details of the two groups before PSM.

Variables		Immediate group (n=219)	Postponed group (n=890)	p
Intervals between OPU and FET (days)		30.77 ± 3.81	80.72 ± 26.49	<0.01
Baseline characters				
Female age (years)		30.84 ± 4.89	31.99 ± 4.77	<0.01
Male age (years)		32.44 ± 5.61	33.81 ± 5.47	<0.01
Female BMI (kg/m^2^)		21.72 ± 2.88	21.94 ± 2.89	0.30
Duration of Infertility (years)		2.93 ± 2.13	3.01 ± 2.40	0.65
AMH (ng/ml)		4.99 ± 4.03	4.43 ± 3.41	0.06
Causes of infertility (n, %)	Tubal factor	86 (39.3)	422 (47.4)	0.15
	Male factor	48 (21.9)	158 (17.8)	
	Anovulation	51 (23.3)	175 (19.7)	
	Endometriosis	8 (3.7)	46 (5.2)	
	UEI	26 (11.9)	89 (10.0)	
COH process				
COH protocols (n, %)	GnRH antagonist	163 (74.4)	690 (77.5)	0.62
	Mild stimulation	49 (22.4)	176 (19.8)	
	PPOS	7 (3.2)	24 (2.7)	
Total doses of Gn (IU)		2279.17 ± 779.96	2350.79 ± 784.10	0.22
Medication of triggering (n, %)	HCG	65 (29.7)	228 (25.6)	0.14
	GnRH agonist	59 (26.9)	289 (32.5)	
	Dual trigger	95 (43.3)	373 (41.9)	
COH duration		9.57 ± 1.39	9.45 ± 1.56	0.28
Trigger day	E_2_ (pg/ml)	3160.57 ± 1387.81	3224.29 ± 1449.60	0.55
	P (ng/ml)	0.99 ± 0.57	1.03 ± 0.67	0.33
No. of oocyte retrieved		12.06 ± 7.12	11.35 ± 6.85	0.17
Type of fertilization (n, %)	IVF	144 (65.8)	620 (69.7)	0.26
	ICSI	75 (34.2)	270 (30.3)	
FET process and outcome				
Endometrium protocols (n, %)	HRT	205 (93.6)	560 (62.9)	<0.01
	OS	3 (1.4)	85 (9.6)	
	NC	11 (5.0)	245 (27.5)	
Stage of embryos transferred (n, %)	Cleavage embryo	134 (61.2)	662 (81.6)	<0.01
	Blastocyst	85 (38.8)	228 (18.4)	
Type of embryos transferred (n, %)	Single	141 (64.4)	433 (48.7)	<0.01
	Double	78 (35.6)	457 (51.3)	
No. of embryos transferred		1.36 ± 0.49	1.51 ± 0.51	<0.01
No. of top-quality embryos transferred		1.28 ± 0.50	1.40 ± 0.55	<0.01
Endometrial thickness (mm)		9.98 ± 1.98	10.01 ± 2.04	0.84
Implantation rate (n, %)		144/297 (48.5)	592/1344 (44.0)	0.16
Clinical pregnancy rate (n, %)		126 (57.5)	506 (56.9)	0.85
Miscarriage rate		20 (15.9)	66 (13)	0.41
Live birth rate (n, %)		105 (47.9)	431 (48.4)	0.89

OPU, oocyte pickup; AMH, anti-müllerian hormone; UEI, unexplained infertility; COH, controlled ovarian hyperstimulation; HRT, hormone replacement therapy; OS, ovulation stimulation; NC, natural cycle; PPOS, progesterone-priming ovarian stimulation.

Both women and men were younger in the immediate group (30.84 ± 4.89 years, 32.44 ± 5.61 years, respectively) compared with the postponed (31.99 ± 4.77 years, 33.81 ± 5.47 years, *P* < 0.05). A higher proportion of patients in the immediate group (93.6%) used HRT than in the postponed group (62.9%, *P* < 0.05). More patients in the immediate group adopted single embryo transfers (64.4%) and blastocyst transfers (38.8%) compared to the postponed one (48.7% and 18.4%, *P* < 0.05). Additionally, the average number of top-quality embryos transferred was significantly lower in the immediate group (1.28 ± 0.50) than in the postponed group (1.40 ± 0.55, *P <*0.05). The clinical pregnancy rate (CPR, 57.5% vs. 56.9%) and LBR (47.9% vs. 48.4%) were comparable between the two groups, respectively (*P >*0.05).

**Figure 1:**
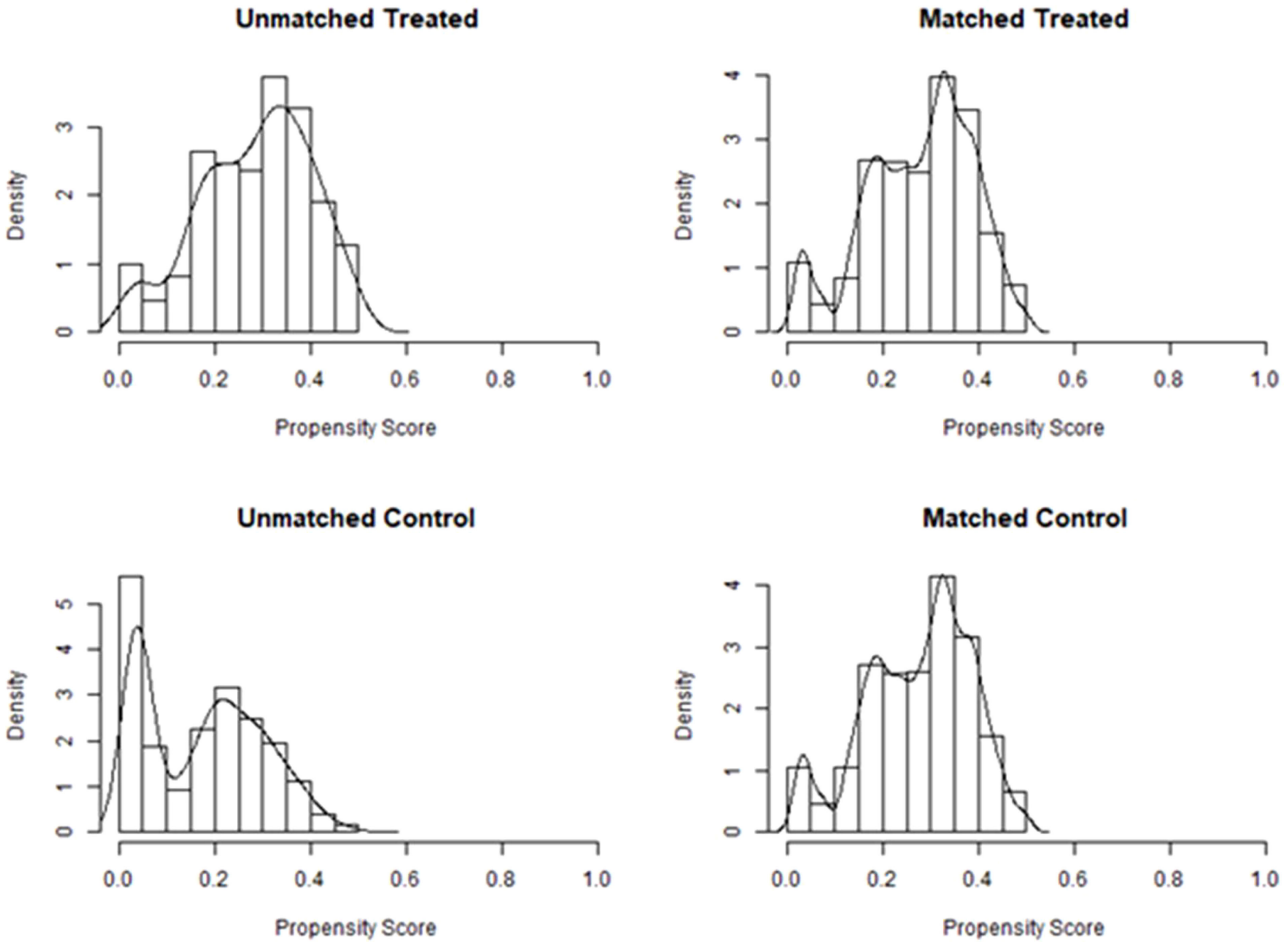
Propensity score before and after matching of the two groups. Treated, the postponed group; Control, the immediate group.

**Figure 2:**
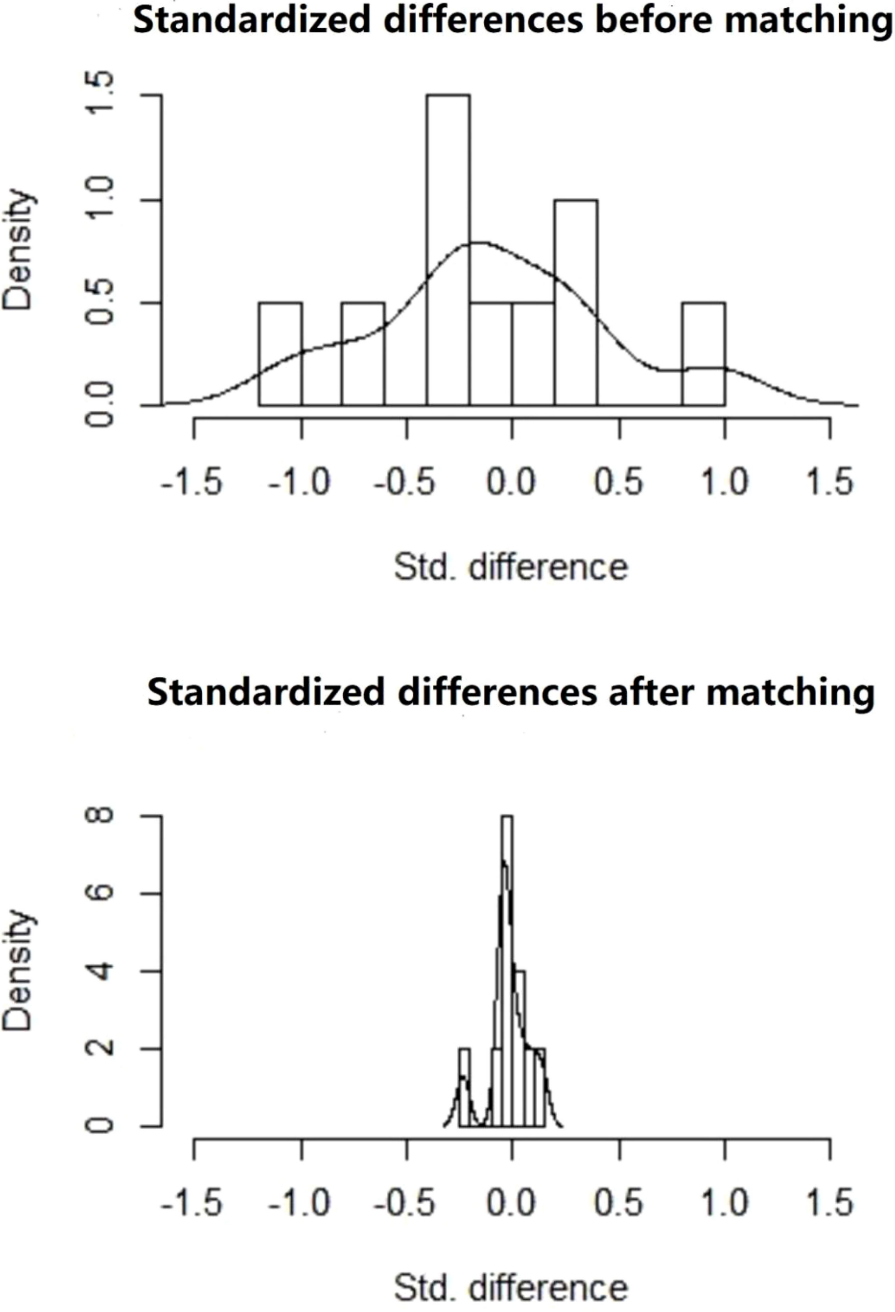
Standardized differences before and after matching.

After PSM, there were 209 patients in the immediate group and 499 patients in the postponed one (see **Table 2**). The female and male age, proportion of primary infertility, endometrial preparation regimen, number and stage of embryos transferred, and the average number of top-quality embryos transferred were all comparable between the two groups. The CPR and LBR in the immediate group were 58.4% and 48.3%, respectively, which were comparable to those of the postponed one (58.1% and 49.7%, *P >*0.05). **Figure 1** shows the propensity score of the two groups before and after matching. **Figure 2** shows the distribution of standardized differences before and after matching.

**Table 2:** Baseline characters and treatment details of the two groups after PSM.

Variables		Immediate group (n=209)	Postponed group (n=499)	p
Intervals between OPU and FET (days)		30.74 ± 3.85	80.39 ± 26.25	<0.01
Female age (years)		31.06 ± 4.85	31.54 ± 4.62	0.21
Male age (years)		32.56 ± 5.68	33.27 ± 5.13	0.11
Female BMI (kg/m^2^)		21.71 ± 2.88	22.01 ± 2.95	0.24
Duration of Infertility (years)		2.91 ± 2.13	3.15 ± 2.51	0.23
AMH (ng/ml)		4.99 ± 4.10	4.93 ± 3.66	0.83
Causes of infertility (n, %)	Tubal factor	81 (38.8)	216 (43.3)	0.07
	Male factor	47 (22.5)	79 (15.8)	
	Anovulation	49 (23.4)	134 (26.9)	
	Endometriosis	6 (2.9)	26 (5.2)	
	UEI	26 (12.4)	44 (8.8)	
COH process				
COH protocols (n, %)	GnRH antagonist	155 (74.2)	382 (76.6)	0.79
	Mild stimulation	47 (22.5)	102 (20.4)	
	PPOS	7 (3.3)	15 (3.0)	
COH duration (days)		9.54 ± 1.40	9.55 ± 1.60	0.97
Medication of triggering (n, %)	HCG	63 (30.1)	125 (25.1)	0.07
	GnRH agonist	55 (26.3)	174 (34.9)	
	Dual trigger	91 (43.5)	200 (40.1)	
Total doses of Gn (IU)		2283.67 ± 782.71	2301.85 ± 809.85	0.68
Trigger day	E_2_ (pg/ml)	3142.43 ± 1384.97	3343.72 ± 1472.91	0.10
	P (ng/ml)	0.96 ± 0.48	1.05 ± 0.69	0.08
No. of oocyte retrieved		11.85 ± 6.65	12.05 ± 731	0.17
Type of fertilization (n, %)	IVF	138 (66.0)	347 (69.5)	0.36
	ICSI	71 (34.0)	152 (30.5)	
FET process and outcome				
Endometrium preparation protocols (n, %)	HRT	195 (93.3)	455 (91.2)	0.64
	OS	3 (1.4)	10 (2.0)	
	NC	11 (5.3)	34 (6.8)	
Stage of embryos transferred (n, %)	Cleavage embryo	134 (64.1)	343 (68.7)	0.23
	Blastocyst	75 (35.9)	156 (31.3)	
No. of embryos transferred (n, %)	Single	130 (62.2)	271 (54.3)	0.09
	Double	79 (37.8)	228 (45.7)	
No. of embryos transferred		1.39 ± 0.48	1.45 ± 0.50	0.08
No. of top-quality embryos transferred		1.31 ± 0.51	1.34 ± 0.56	0.52
Endometrial thickness (mm)		10.01 ± 1.97	9.95 ± 1.94	0.71
Implantation rate (n, %)		140/288 (48.6)	335/727 (46.1)	0.47
Clinical pregnancy rate (n, %)		122 (58.4)	290 (58.1)	0.95
Miscarriage rate		20/122 (16.4)	38/290 (13.1)	0.38
Live birth rate (n, %)		101 (48.3)	248 (49.7)	0.74

OPU, oocyte pickup; AMH, anti-müllerian hormone; UEI, unexplained infertility; COH, controlled ovarian hyperstimulation; HRT, hormone replacement therapy; OS, ovulation stimulation; NC, natural cycle; PPOS, progesterone-priming ovarian stimulation.

**Table 3** shows the neonatal outcomes before and after PSM. A total of 105 patients and 431 patients took babies home in the immediate and the postponed groups, respectively. The average birth weight, birth height, gestational age, proportion of twin babies, sex ratio, the proportion of premature delivery, macrosomia, and low birth weight, the incidence of pregnancy related complications were all comparable between the two groups. After matching, no significant difference was found in the neonatal outcomes between the two groups either.

**Table 3:** Neonatal outcomes of the two groups.

Variables		Before PSM			after PSM		
		Immediate group (n=105)	Postponed group (n=431)	P	Immediate group (n=101)	Postponed group (n=248)	P
Gestational age (day)		268.86 ± 15.78	270.55 ± 29.36	0.57	261.83 ± 72.29	268.91 ± 32.45	0.18
Birth weight (g)		3087.24 ± 591.78	3012.48 ± 624.73	0.31	3088.82 ± 565.35	3038.64 ± 625.78	0.52
Birth height (cm)		49.41 ± 2.11	49.15 ± 2.52	0.29	49.08 ± 1.87	49.30 ± 2.52	0.89
Preterm birth (n, %)		18 (17.1)	77 (17.9)	1.0	18 (17.8)	43 (17.3)	0.91
Singleton (n, %)		88 (83.8)	338 (78.4)	0.28	85 (84.2)	200 (80.6)	0.44
Twins (n, %)		17 (16.2)	93 (21.6)		16 (15.8)	48 (19.4)	
Macrosomia (n, %)		6/122 (4.9)	28/524 (5.3)	1.00	6/117 (5.1)	17/296 (5.7)	0.82
Low birthweight (n, %)		18/122 (14.8)	89/524 (17.0)	0.58	17/117 (14.5)	28/296 (9.5)	0.14
Gender (n, %)	Male	63/122 (51.6)	270/524 (51.5)	1.0	61/117 (52.1)	143/296 (48.3)	0.48
	Female	59/122 (48.4)	254/524 (48.5)	56/117 (47.)	153/296 (51.7)
Pregnancy complications (n, %)	
		4 (3.8)	10 (2.9)		3 (3.0)	5 (2.0)	0.59

## Discussion

After implementation of the freeze-all policy, one of the most common concerns of patients is when they can perform FET. From the viewpoint of clinicians, the influence of the previous fresh cycles, the patient’s physical and psychological conditions, and the clinical outcomes of FET should all be taken into account to make a best decision.

It has long been established that the gene profile involved in normal endometrial receptivity during COH is altered compared with that of natural cycles (16–21). Consequently, the freeze-all strategy was advocated to circumvent the disadvantages of fresh embryo transfer (6, 22). A new question arises that how long the patients should wait until the negative effects of COH are fading out. Some studies showed that delayed FET performed following a failed fresh embryo transfer did not increase or decrease CPR (23, 24). However, results from another two randomized controlled trials published in 2021 showed that an immediate FET could yield a better CPR (25, 26).

Regarding the freeze-all strategy, the optimal interval to perform FET is still contentious. Studies published from 2016 to 2018 found that performing FETs within the first menstrual cycle following OPU or afterward did not affect LBR (8, 27, 28). However, the inherent bias of these retrospective studies weakened the power of evidence. In 2019, Huang et al. and Higgins et al. employed a PSM model to address this issue and found that FET performed within the first menstrual cycle was associated with a higher CPR and LBR than delayed FET (7, 9). However, both studies ignored the embryo quality, one of the most important factors related to CPR, thus making their conclusions less convincing. Moreover, both studies failed to propose a reasonable explanation for the better clinical outcomes of immediate FET within the first menstrual cycle.

Our study also adopted a PSM model to explore the effect of length of waiting period on clinical outcomes. The propensity score (PS) method itself cannot control confounding factors, but it can improve the balance between the groups by PS matching, weighting, stratification, or covariate adjustment to achieve the effect of “ randomization”, also known as post-randomization. Briefly, PSM is used to match patients with a similar distribution of confounders so that the difference in outcomes gives an unbiased estimate of the treatment effect (29). Before matching, we could see that patients were younger in the immediate group, suggesting that they might have a higher reproductive potential, and thus a higher proportion of single embryo and blastocyst transfers were carried out in this group. Due to the fact that ovulation was rarely occurred in the first menstruation after OPU, HRT was used by 93.6% of patients in the immediate group, leading to a disequilibrium in endometrial preparation regimens between the two groups. Additionally, in our analysis, we considered the impact of embryo quality, which was neglected by the two previous studies (7, 9). It is well-known that embryo quality is associated with developmental potential, which greatly impacts CPR (30, 31). Before matching, the average number of top-quality embryos transferred was also significantly different between the two groups (1.40 ± 0.55 vs. 1.28 ± 0.50, *P <*0.05). However, these unbalanced variables were all adjusted by the PSM model. Unlike the previous two studies that used a matching ratio of 1:1, we adopted a ratio of 1:4, which minimized the loss of sample size. The results after PSM suggested that the COH process did not influence the endometrial receptivity in subsequent FET cycles and a normal endometrial environment may be restored after only 1 menstrual cycle. In our study, an immediate FET did not improve nor worsen the clinical outcome, but it shortened the time patients wait to be pregnant successfully, which alleviated their anxiety compared with those in the postponed FET group. Our results were consistent with two recently published meta-analysis that did not find any association between the timing of FET and pregnancy outcomes (11, 32).

Apart from the clinical outcomes, another concern is the health of babies conceived by FET. A growing number of studies have shown that FET is associated with an increased risk of high birth weight and macrosomia (33–36). Does the length of interval between OPU and FET affects neonatal outcomes? Two recently published studies explored this issue but ended up with conflicting results (12, 13). A study by Hu et al. carried out single blastocyst transfers in patients and found that postponing FET was associated with an increased risk of macrosomia (13). They attributed the observation to the extended freezing time in the postponed group. Actually, in their study, the interval between OPU and FET more than 40 days were all classified as the postponed group. The upper limit of the interval was not mentioned. By contrast, we only included patients performed FET within 6 months after OPU into the postponed group, and so did He et al. (12). They found that the average birth weight and height, sex ratio, and the ratio of macrosomia and low birth weight did not differ significantly between the immediate and postponed FET groups, which was consistent with our results. These results indicated that a delay of up to 6 months after OPU to perform FET didn’t impact the birth weight, but the influence of delaying longer than 6 months needed further exploration.

In our study, when to perform FET after OPU was mostly decided by patients themselves, thus the basic characteristics and treatment processes were uneven between both groups, which is inevitable in a retrospective study. Though we can say that we carried out a real-world study using a PSM model to control confounding factors, the lack of randomization still undercuts the statistical power of the study. More prospective studies are needed to address this issue.

In conclusion, we found that immediate FET effectively saved the time to pregnancy for patients and also yielded similar clinical and neonatal outcomes compared with the postponed group. Therefore, if there are no contradictions and patients wish to be pregnant as soon as possible, FET should be performed immediately after OPU.

## Data availability statement

The raw data supporting the conclusions of this article will be made available by the authors, without undue reservation.

## Ethics statement

The studies involving human participants were reviewed and approved by the ethics committee of the Zhejiang Provincial People’s Hospital. The patients/participants provided their written informed consent to participate in this study.

## Author contributions

All authors contributed to the study. Study conception and design, data collection, analysis and manuscript writing were performed by MJ. BJ contributed significantly to data analysis; XG, RW, and YJ contributed to clinical practice and follow-up; LZ supervised the whole study, helped the original manuscript revision and data analysis; JS helped manuscript revision. MJ obtained fund and supported this research. All authors have read and approved the final manuscript.

## Funding

This work was supported by basic public welfare research program of Zhejiang Province of China (Grant Number: LGF20H040012), medical and health clinical research project of Zhejiang Province of China (Grant Number:2021KY525) and adjunct talent fund of Zhejiang Provincial People’s Hospital.

## Acknowledgments

We thank the patients whose data were used in this study. Furthermore, we thank TopEdit (www.topeditsci.com) for its linguistic assistance during the preparation of this manuscript.

## Conflict of interest

The authors declare that the research was conducted in the absence of any commercial or financial relationships that could be construed as a potential conflict of interest.

## Publisher’s note

All claims expressed in this article are solely those of the authors and do not necessarily represent those of their affiliated organizations, or those of the publisher, the editors and the reviewers. Any product that may be evaluated in this article, or claim that may be made by its manufacturer, is not guaranteed or endorsed by the publisher.
